# Charge Injection Characteristics of Semi-Conductive Composites with Carbon Black-Polymer for HVDC Cable

**DOI:** 10.3390/polym11071134

**Published:** 2019-07-03

**Authors:** Yanhui Wei, Mingyue Liu, Wang Han, Guochang Li, Chuncheng Hao, Qingquan Lei

**Affiliations:** 1 Institute of Advanced Electrical Materials, Qingdao University of Science and Technology, Qingdao 266042, China; 2 State Key Laboratory of Electrical Insulation and Power Equipment, Xi’an Jiaotong University, Xi’an 710049, China

**Keywords:** semi-conductive composites, carbon black-polymer, charge injection characteristics, HVDC cable

## Abstract

Semi-conductive composites composed of carbon black-polymer play an important role in uniform electric field in high voltage direct current (HVDC) cable. They also affect space charge behaviors in the insulation material. However, the charge injection characteristics of semi-conductive composites are not detailed. In this work, the electrode structure of ‘Semi-conductive composites- Insulation material- Metal bottom’ (S-I-M) is proposed, and the currents formed by injected charges from semi-conductive composites are characterized by the thermally stimulated depolarization current (TSDC) method. Further, the experimental results based on the structure of S-I-M are compared with the traditional electrode structure of M-I-M (Metal upper electrode- Insulation material- Metal bottom electrode) and the simplified cable electrode structure of MS-I-M (Metal upper electrode-Semi-conductive electrode- Insulation material- Metal bottom electrode), respectively. The experimental results show that the semi-conductive composite plays an important role in the charge injection process and it presents a different tendency under different compound modes of temperature and electric field. For the low electric field (*E* ≤ 5 kV/mm) and the low temperature (*T* ≤ 50 °C), the current caused by the accumulated charges follows the rule, *I*_S_ > *I*_MS_ > *I*_M_. For the low electric field and high temperature (*T* > 50 °C), the current caused by the injected charges follows the rule, *I*_MS_ > *I*_M_ > *I*_S_. This phenomenon is closely related to the interface characterization and contact barrier.

## 1. Introduction

The semi-conductive layer is an essential component of high voltage direct current (HVDC) cables and plays an important role in the uniform electric field and makes the conductor wire core and the insulation layer connect tightly [1,2,3,4,5,6,7]. The semi-conductive composites are mainly composed of ethylene-vinyl acetate copolymer (EVA), low-density polyethylene (LDPE) and carbon black (CB). In the actual application, space charge accumulation in the insulation layer is one of the key factors that threaten the safe operation of the HVDC cable, which can cause local electric field distortion, resulting in the degradation or breakdown of the insulation material. The semi-conductive layer is located between the conductor wire core and the insulation layer. As the electron transport path from the conductor to the insulation material, it affects space charge accumulation in the insulation material [8,9,10,11,12,13]. Hence, it is important to thoroughly understand the charge injection characteristic of semi-conductive composites.

The space charge problem in the cable insulation material has been investigated by theoretical calculation and experimental measurements in many works [1,14,15,16,17]. However, most studies ignored the effect of charges injected by the semi-conductive layer, only focusing on the injected charges from the metal electrode, such as the thermally stimulated depolarization current (TSDC), pulsed electroacoustic (PEA) and space charge limited current (SCLC).

In recent years, there have been some studies paying attention to the semi-conductive composites of the HVDC cable mainly from two aspects: the interfacial characterization between the semi-conductive composites and the insulating material, and the modification of semi-conductive material [2,11,14,18,19,20,21,22,23]. For the former, some works have been investigated, including the aging mechanism of the interface between the semi-conductive composites and the insulating material under stress of high voltage, and the relationship between breakdown field strength and insulation structure nearby the semi-conductive composites [2,11,14]. For the latter, the influence of the doping ratio of carbon black on the conductivity of the semi-conductive composites has been studied as well as the influence of semi-conductive prescription on the space charge behaviors of the insulation material [18,19,20,21,22,23].

The above studies all tried to solve the space charge accumulation in the insulation material in the view of the space charge source. However, the injected charges were considered to come entirely from the metal electrode, ignoring the charges injected by semi-conductive material. At present, the charge injection characteristic of the semi-conductive composites in the HVDC cable are not detailed as it is difficult to distinguish the charge injection from the metal electrode and semi-conductive composites. Hence, it is not clear what the contribution of the space charge from the semi-conductive composite is.

In this work, in order to analyze the charge injection characteristics of the semi-conductive composites, the electrode structure of the semi-conductive composites is designed as a high voltage terminal. Then, the currents formed by charges injected by the semi-conductive composites are characterized by the thermally stimulated depolarization current (TSDC) method. Further, the experimental results based on the structure of S-I-M (Semi-conductive composites- Insulation material- Metal bottom) are compared with the traditional electrode structure of M-I-M (Metal upper electrode- Insulation material- Metal bottom electrode) and the simplified cable electrode structure of MS-I-M (Metal upper electrode-Semi-conductive electrode- Insulation material- Metal bottom electrode), respectively. Finally, the charge injection mechanisms with different high voltage terminals are discussed.

## 2. Experimental Method and Setup

### 2.1. Preparation of Semi-Conductive Composite

**Figure 1:**
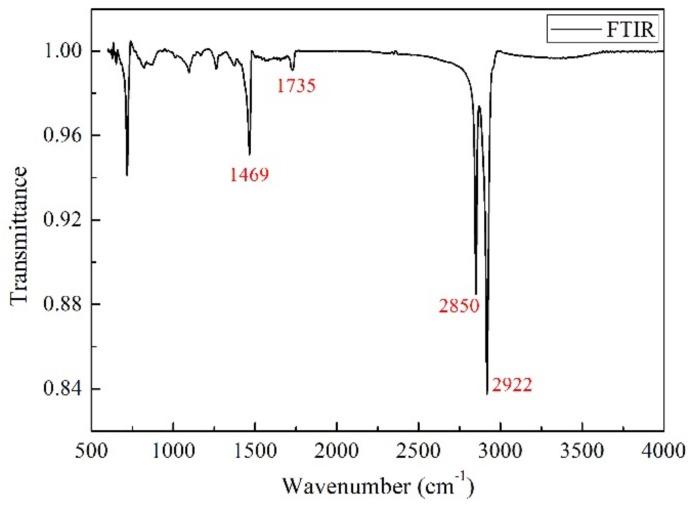
Fourier transform infrared spectroscopy (FTIR) image of semi-conductive composites.

For the HVDC cable, the semi-conductive layer is a kind of composite, which mainly includes ethylene-vinyl acetate copolymer (EVA), low-density polyethylene and carbon black (CB). The insulation layer is mainly composed of polyethylene (PE), a cross-linking agent. Fourier transform infrared spectroscopy (FTIR) of the semi-conductive composite was implemented is shown in Figure 1.

The results show that there are four typical absorption peaks at 1469 cm^−1^, 1735 cm^−1^, 2850 cm^−1^ and 2922 cm^−1^. As can be seen from Figure 1, a strong absorption peak appears near 2922 cm^−1^ and 2985 cm^−1^, which are both characteristic absorption peaks of the stretching vibration of –CH_2_ in PE. The absorption peak near 1735 cm^−1^ is weak, which is the characteristic peak of –CO stretching vibration in CB or EVA. The absorption peak at 1469 cm^−1^ is the –CH telescopic vibration peak in PE. The absorption peaks less than 1000 cm^−1^ represent the vibrations of small molecular groups, which are not analyzed here [24,25].

**Figure 2:**
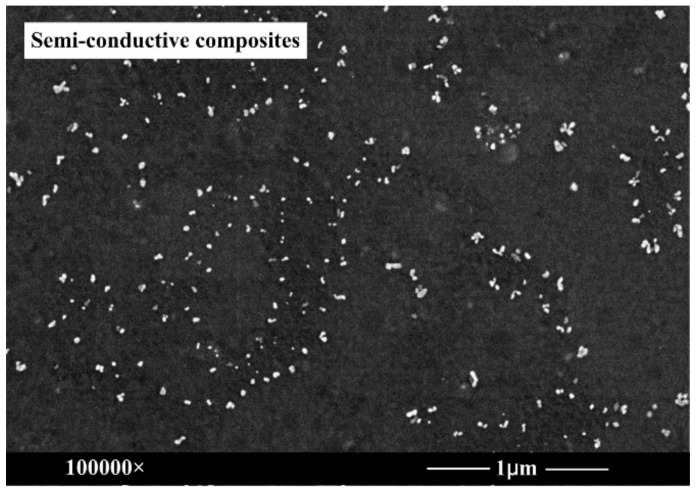
Scanning electron microscopy (SEM) image of semi-conductive composites.

**Figure 3:**
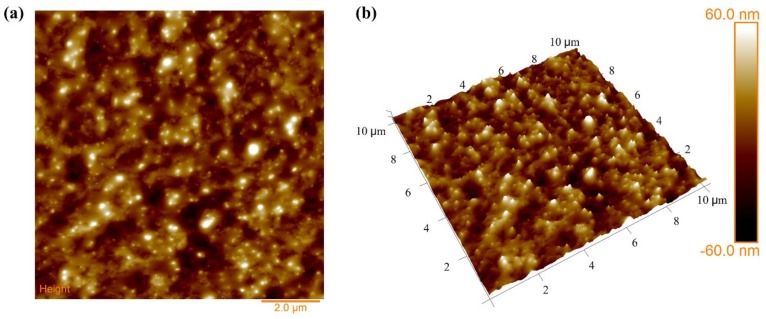
Atomic force microscopy (AFM) image of semi-conductive composites. (**a**) Two-dimensional distribution; (**b**) three-dimensional distribution.

In the experiments, the semi-conductive composites with a thickness of 200 μm were prepared by the melt-compounding method. Firstly, the raw materials were dried in a vacuum chamber at 60 °C for 2 h, and were then molded by a plate vulcanizing press at 110 °C for 10 min. After that, the cross linked process was implemented by another plate vulcanizing press at 180 °C for 15 min. Finally, the specimen was cooled for 10 min. Adopting a similar method, cross-linked polyethylene (XLPE) films with a thickness of 300 μm were prepared. The dispersion properties of carbon black particles in the polymer matrix were observed by scanning electron microscopy (SEM) and the surface roughness of composites was observed by atomic force microscopy (AFM), as shown in Figure 2 and Figure 3.

As seen in Figure 2, carbon black particles and white spots were observed clearly. They filled in the polymer matrix, forming the electron conductive channel. Overall, most carbon black particles were dispersed uniformly in the polymer matrix. The surface roughness of the semi-conductive composites had a great effect on the charge emission characteristic, because semi-conductive composites are composed of countless tiny conductive spheres, which can cause the interface electric field distortion between the semi-conductive layer and the insulation layer. From Figure 3a, it can be seen that the average roughness of the semi-conductive composites was 11.9 nm.

### 2.2. Test Method and Validation

**Figure 4:**
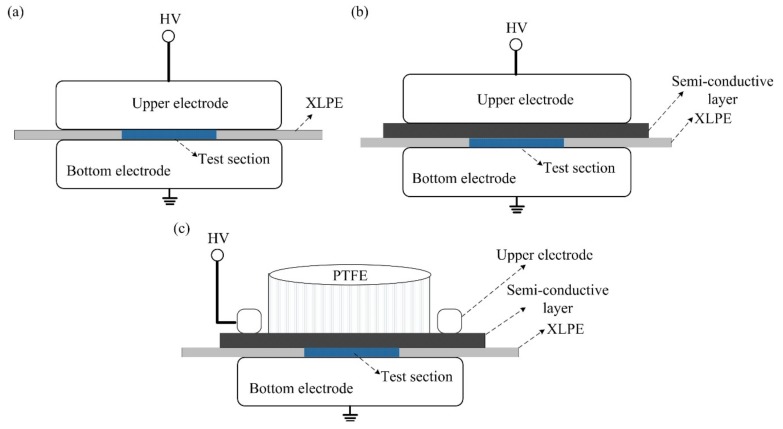
Schematic diagram of electrode structures with different high voltage terminals. (**a**) The traditional electrode structure of Metal upper electrode- Insulation material Metal bottom electrode (M-I-M); (**b**) the simplified cable electrode structure of Metal upper electrode-Semi-conductive electrode- Insulation material- Metal bottom electrode (M-S-I-M); (**c**) the electrode structure of Semi-conductive composites- Insulation material- Metal bottom (S-I-M).

In most studies, the traditional electrode structure of ‘Metal upper electrode- Insulation material- Metal bottom electrode’ (M-I-M) is adopted to study the conductivity property and charge property of the insulation material, as shown in Figure 4a, in which the metal of the high voltage terminal applies voltage on the insulation material. In the actual application, the cable mainly consists of a metal wire core, an inner semi-conductive layer, an insulation layer, an outside semi-conductive layer and a metal armor layer. Among them, the inner semi-conductive layer between the metal wire core and the insulation layer plays an important role in the space charge accumulation property in the insulation layer. Figure 4b shows the simplified cable electrode structure of ‘Metal upper electrode-Semi-conductive electrode- Insulation material- Metal bottom electrode’ (M-S-I-M), in which the metal and semi-conductive layer are a whole high voltage terminal applying voltage on the insulation material. While, it is difficult to distinguish whether the charges accumulated in the insulation material come from the metal electrode or the semi-conductive electrode.

In this work, a kind of electrode structure for assessing the charge emission characteristics of semi-conductive composites was proposed, in which the semi-conductive composites acted as a high voltage terminal applying voltage on the insulation material, as shown in Figure 4c.

**Figure 5:**
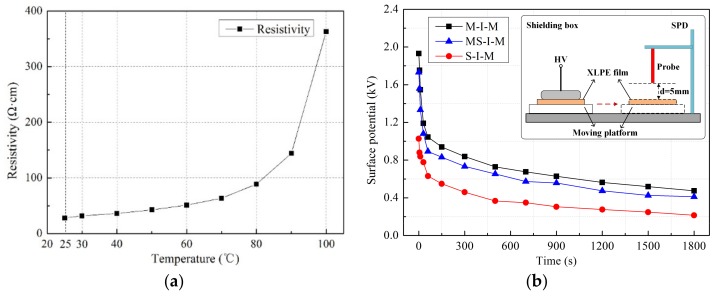
Test method and validation. (**a**) Changes of resistivity of semi-conductive composites with temperature; (**b**) surface potential decay over time of semi-conductive composites under different electrode structures.

For the method, a specific ring structure made of aluminum was designed as the upper electrode, which was connected closely with the semi-conductive layer composed of EVA, LDPE and CB. When applying voltage on the ring structure, the equal potential formed on the semi-conductive composites because the resistivity of semi-conductive composites was much lower than that of the insulation material. Figure 5a shows changes of the resistivity of the semi-conductive composites with temperature. It can be seen that the volume resistivity changes from 25 Ω·cm to 85 Ω·cm at the test temperature from 25 °C to 80 °C. While, the volume resistivity of XLPE is around 10^16^ Ω·cm. In order to ensure the charge emission was from the semi-conductive composites rather than the ring structure, the test region of the specimen was much smaller than the ring structure, which is marked in blue in Figure 4c, and the diameter of the test region was 20 mm. Besides, in order to make a good contact between the semi-conductive composites and the specimen, an insulation structure of cylinder made of heat-resisting material of polytetrafluoroethylene (PTFE) with the same weight of the upper electrode in Figure 4a,b was placed on the semi-conductive composites.

In the experiments, the charge injection processes with different high voltage terminals were firstly carried out based on the three-electrode structure. After that, the accumulated charges in the insulation material injected by different high voltage terminals were characterized by thermally stimulated depolarization current (TSDC), which can reflect the changes of the trapped charges in insulation material with the increasing depolarization temperature. For the charge injection processes, three typical polarization temperatures of 25 °C, 50 °C and 70 °C were considered, two typical electric fields of 5 kV/mm and 60 kV/mm were set and four typical compound modes of temperature and electric field were carried out, that is, low temperature and high field, high temperature and low field, low temperature and low field, high temperature and high field. For each compound mode, the electrode structure of S-I-M was placed in a temperature control chamber, and the applying voltage time was 30 min. For the TSDC process, the specimen was taken out at room temperature for the depolarization current measurement. The depolarization temperature changed from 25 °C to 80 °C, the heating rate was 2 °C/min. Keeping the same specimen condition and test method, the traditional electrode structure of M-I-M and the simplified cable electrode structure of MS-I-M were carried out.

In order to verify the test method, surface potential decay curves of XLPE stressed of different electrode structures were measured by a non-contact surface potentiometer. In theory, the injected charges in the insulation material are different for different high voltage terminals, and the changes can be reflected by the surface potential, which decays over time after the voltage is removed. Firstly, the specimen was applied voltage under 15 kV/mm at 25 °C and the duration of the voltage was 30 min. Secondly, after charging, the specimen was moved instantly to the measuring position via a moving platform. During the measurement, the distance between the probe and XLPE film was set to 5 mm. The surface potential was recorded for 30 min. The measured results are shown in Figure 5b.

It can be seen that the initial surface potential was different for the three types of electrode structures. The maximum surface potential was 1.93 kV caused by the electrode structure of M-I-M, followed by the electrode structure of MS-I-M with 1.73 kV and S-I-M with 1.03 kV. It indicated that there was an obvious charge injection of the semi-conductive composites. It should be noted that the surface potential decay (SPD) method was only used to verify qualitatively the charge emission characteristics with different electrode structures, because it only reflects the changes of surface charge. In the experiments, the accumulated charges in the insulation material bulk were characterized by the TSDC method.

## 3. Experimental Results and Analysis

**Figure 6:**
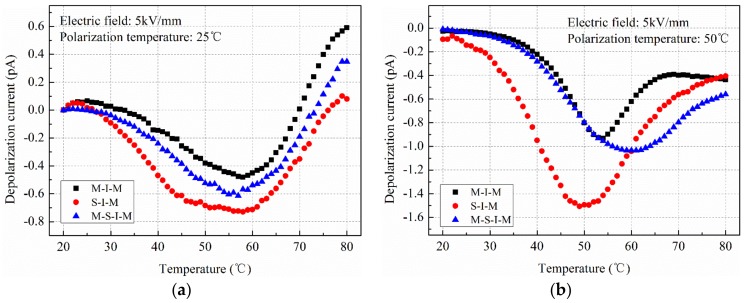

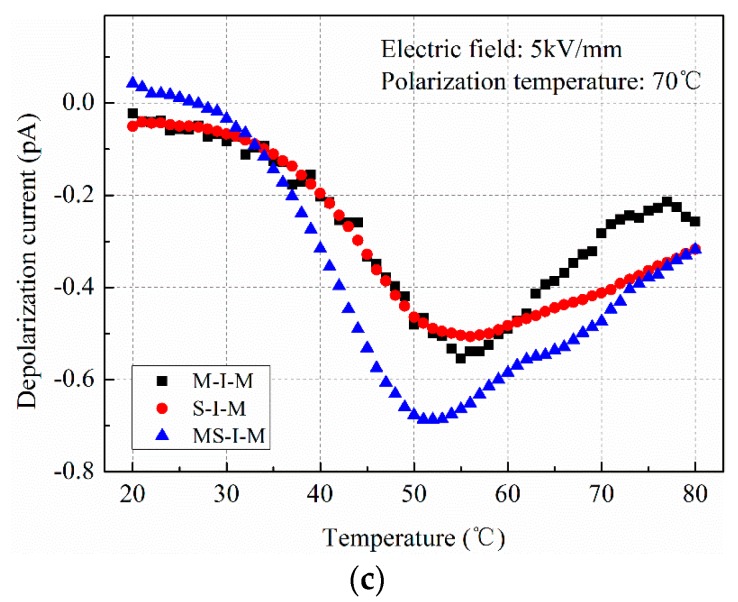
Depolarization current of cross-linked polyethylene (XLPE) polarized using different electrode structures under low electric field with different polarization temperatures. (**a**) 25 °C; (**b**) 50 °C; (**c**) 70 °C.

In the experiments, two typical electric fields, a low electric field of 5 kV/mm and a high electric field of 60 kV/mm, were considered. Three typical polarization temperatures of 25 °C, 50 °C and 70 °C were set and the depolarization temperature was changed from 25 °C to 80 °C, the heating rate was 2 °C/min. Figure 6 shows the depolarization current of XLPE polarized using three types of electrode structures under the low electric field.

Generally, depolarization currents of dielectric mainly have contribution from the dipole depolarization process and de-trapping process of the captured charges [26]. For different electrode structures, dipole depolarization processes are the same because the same voltages are applied on the insulation material. While, the charge injection processes are different, these charges will struggle gradually against the trap centers with the increasing temperature. Hence, the depolarization current can represent the injected charges from different high voltage terminals. As shown in Figure 6, for the three electrode structures, the depolarization current firstly increases, and then decreases with the increasing depolarization temperature, and the maximum current appears at around 50 °C to 60 °C. Besides, the maximum depolarization currents made changes when polarization temperatures were different for the three electrode structures, which illustrate the effects of temperature on the injected charge from the metal electrode and semi-conductive electrode are different. When the polarization temperature is lower than 50 °C, the injected charges from the semi-conductive electrode are obviously larger than that from the metal electrode. It illustrates that the semi-conductive composites play a great significant role in the charge injection process, as shown in Figure 6a,b. The maximum current caused by the injected charges from the semi-conductive composites was 0.8 pA at 25 °C, and the value increased to 1.51 pA at 50 °C. When the polarization temperature exceeded 50 °C, it was found that the charge emission characteristics were different for the three electrode structures. The charges from the electrode structure of MS-I-M were larger than that of M-I-M and S-I-M, the maximum current was 0.69 pA at 70 °C, as shown in Figure 6c.

**Figure 7:**
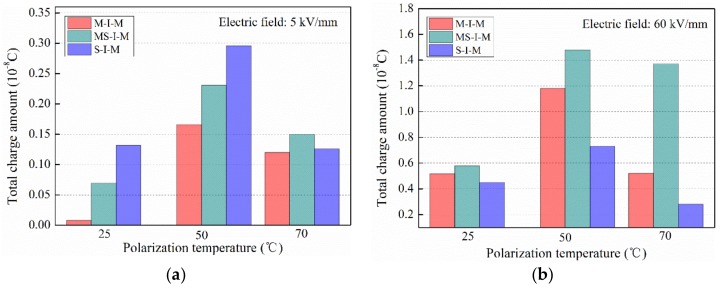
Total charge amount as a function of the polarization temperature in XLPE stressed of different electrode structures. (**a**) Low electric field of 5 kV/mm; (**b**) high electric field of 60 kV/mm.

In the experiments, the insulation specimens polarized under a high electric field of 60 kV/mm were also carried out, adopting the three types of the electrode structures, and the total charges were calculated by the TSDC curves [26]. Figure 7 shows total charge amount as a function of the polarization temperature in XLPE stressed of different electrode structures. Comparing the two cases of low electric field and high electric field, it was observed from Figure 7a that when the polarization temperature was lower than 50 °C, the charges from the semi-conductive composites were obviously larger than that from the metal electrode. When the polarization temperature exceeded 50 °C, the charges from the whole electrodes of ‘MS’ became evident, while the charges from the semi-conductive composites cannot be ignored, since both of them had the same order. The phenomenon was closely related to the interface characterization and the contact barrier among the metal, the semi-conductive composites and the insulation material. It can be observed from Figure 7b that for different polarization temperatures, the total charges all present the consistent variation trend, that is, the charges from the electrode structure of MS-I-M were the most among the three electrode structures. The same phenomenon occurred at the low electric field and high temperature, as shown in Figure 7a.

**Figure 8:**
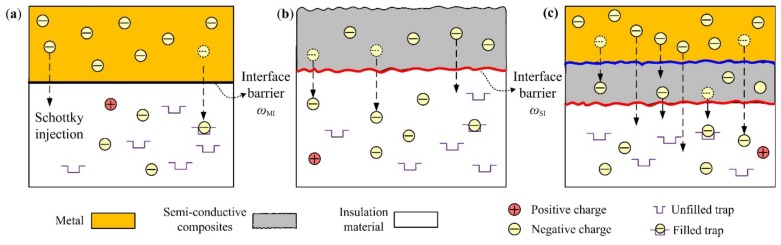
Schematic diagram of charge injection from different high voltage terminals. (**a**) M-I; (**b**) S-I; (**c**) MS-I.

Comparing the three types of electrode structures, it can be summarized that the semi-conductive composite a significant influence on charge accumulation in the insulation material, and for different compound modes of temperature and electric field, the charge injection characteristics from the semi-conductive composites were different. Figure 8 shows a schematic diagram of charge injection from different high voltage terminals. Here, the low electric field refers to how the applied electric field is lower than 5 kV/mm, and the relatively low temperature refers to how the polarization temperature is lower than 50 °C. For the low electric field and the low temperature, the depolarization current follows the rule, *I*_S_ > *I*_MS_ > *I*_M_. For the low electric field and high temperature, the rule becomes *I*_MS_ > *I*_M_ > *I*_S_.

For the low electric field and the low temperature, it was difficult to obtain sufficient energy for the electrons in the metal electrode to overcome the interface barrier between the metal and the insulation material, hence the injected charges from the traditional electrode structure were few, as shown in Figure 8a. For XLPE, the charge injection electric field at the room temperature was around 10 kV/mm [14]. While the charge injection characteristic was different for the semi-conductive composites, which consist of countless tiny carbon black particles (see Figure 2), as shown in Figure 8b. From the AFM image in Figure 3a, it can be seen that the average roughness of the semi-conductive composites was 11.9 nm. These tiny bump structures on the semi-conductive composites surface can cause the interface electric field distortion between semi-conductive composites and the insulation material, resulting in the lower injected barrier. Hence, the charges can be easily injected from the electrode structure of S-I-M under the condition of the low electric field and the low temperature.

For the low electric field and high temperature or the case of the high electric field, the charges from the whole electrode structure of MS-I-M were the largest among three electrode structures, as shown in Figure 8c. This is because most charges in the metal and semi-conductive composites could get enough energy under the effect of temperature and electric field to overcome the interface barrier and inject into the insulation material, especially for the high temperature of 70 °C or the high electric field of 60 kV/mm. By contrast, for the electrode structure of M-I-M, there was no contribution of charges injected by semi-conductive composites. Besides, the interface barrier between the metal and insulation material was higher than that between the semi-conductive composites and the insulation material, which led to the injected charges from the electrode structure of M-I-M being less than that of the electrode structure of MS-I-M. It also implied that the semi-conductive composite plays a key role during the process of charge exchange.

In addition, the maximum charge amount all appears at the polarization temperature of 50 °C, which has a close link with the charge transport of trapping and de-tapping in the insulation material, as shown by the charge transport processes in the insulation material in Figure 8. A similar phenomenon has also been found in LDPE stressed by the metal electrode [26]. At room temperature, it is difficult for the captured charges to release from the trap sites, resulting in the accumulation of homo-charges near the cathode, thus depressing the further injection of the charges. When the temperature exceeds about 50 °C, the charge movement is accelerated which can enhance the hopping probability of charges between the adjacent traps, resulting in less charge accumulation.

## 4. Conclusions

In conclusion, the injection charge characteristics with different high voltage terminals (S-I-M, M-I-M and MS-I-M) were investigated by the thermally stimulated depolarization current method. The conclusions are drawn as follows:

(1) For the low electric field (*E* ≤ 5 kV/mm) and the low temperature (*T* ≤ 50 °C), the current caused by the injected charges follows the rule, *I*_S_ > *I*_MS_ > *I*_M_. The phenomenon can be explained in that it is difficult for the electrons in the metal electrode to overcome the interface barrier between the metal and the insulation material. While, for semi-conductive composites, the charges can be injected to the insulation material because of countless tiny conducting spheres, which can cause the interface electric field distortion between semi-conductive composites and the insulation material, resulting in the lower interface barrier.

(2) For the low electric field (*E* ≤ 5 kV/mm) and high temperature (*T* > 50 °C) or the case of a high electric field, the current caused by the injected charges follows the rule, *I*_MS_ > *I*_M_ > *I*_S_. This can be interpreted as how most charges in the metal and semi-conductive composites can get enough energy to overcome the interface barrier. By contrast, for the electrode structure of M-I-M, there is no contribution of injected charges from semi-conductive composites and the interface barrier between the metal and insulation material is higher than that between the semi-conductive composites and the insulation material.
